# Do the representative beam data for TrueBeam^™^ linear accelerators represent average data?

**DOI:** 10.1002/acm2.12518

**Published:** 2019-01-13

**Authors:** Yoshihiro Tanaka, Hirokazu Mizuno, Yuichi Akino, Masaru Isono, Norimasa Masai, Toshijiro Yamamoto

**Affiliations:** ^1^ Department of Radiation Therapy Japanese Red Cross Society Kyoto Daiichi Hospital Kyoto‐shi, Kyoto Japan; ^2^ Department of Medical Physics and Engineering Osaka University Graduate School of Medicine Suita‐shi, Osaka Japan; ^3^ Oncology Center, Osaka University Hospital Suita‐shi, Osaka Japan; ^4^ Department of Radiation Oncology Osaka International Cancer Institute Osaka‐shi, Osaka Japan; ^5^ Miyakojima IGRT Clinic Osaka‐shi, Osaka Japan; ^6^ Department of Radiology Saiseikai Noe Hospital Osaka‐shi, Osaka Japan

**Keywords:** commissioning, linear accelerator, representative beam data

## Abstract

If the vendor's representative beam data (RBD) for TrueBeam linear accelerators are to be valid for use in clinical practice, the variations in the beam data used for beam modeling must be small. Although a few studies have reported the variation of the beam data of the TrueBeam machines, the numbers of machines analyzed in those studies were small. In this study, we investigated the variation in the beam data for 21 TrueBeam machines collected from 17 institutions with their agreement. In the exponential regions, the percent depth dose (PDD) values showed very small variation, <1% for all the photon energies analyzed. Similarly, the off‐center ratio (OCR) values also showed small variation for all energies. In the field regions, the standard deviations of the values of dose difference (DD) between the data for each machine and the study average were <1% for field sizes ≥100 × 100 mm^2^. The maximum distance‐to‐agreement from the average data was <0.5 mm in the penumbra regions. The output factor (OPF) values also showed very small variation (<1%) for all energies and field sizes. Both the PDD and OCR of the average study data showed good agreement with the vendor's RBD for field sizes ≥100 × 100 mm^2^. The OPF of the average study data also showed good agreement with the vendor's RBD for all field sizes. However, although all the institutions used ionization chambers with similar cavity volumes, the 30 × 30 mm^2^ field size showed large DD variations (≥2%) in OCR in the field regions. We conclude that the intermachine variability of TrueBeam linear accelerators was very small except for small field dosimetry, supporting the validity of the use of the RBD for clinical applications. The use of the vendor's RBD might greatly facilitate the quick installation of a new linear accelerator.

## 1 INTRODUCTION

Improvements to radiotherapy treatment planning systems (TPSs) have enabled the development of advanced radiotherapy techniques such as intensity‐modulated radiotherapy,^1^ volumetric‐modulated arc therapy,^2^ and stereotactic radiotherapy.^3^ Accuracy of beam modeling and in the commissioning of TPSs is essential for these procedures.^4^ However, a credentialing study of radiation oncology centers in the USA reported that roughly 30% of the institutions failed to deliver the dose distribution specified in the TPS to a head and neck phantom.^5^ According to the World Health Organization Radiotherapy Risk Profile,^6^ approximately one‐fourth of adverse events in radiotherapy are associated with the commissioning of the TPS.

Many institutions tend to base their beam modeling in the TPS on machine data provided by the vendor of their linear accelerator, for example, the Halcyon released recently by Varian Medical Systems (Palo Alto, CA, USA). However, these data may be inadequate for clinical use because the beam characteristics can differ significantly between machines because of slight variations in design, changes that occur during installation, and beam tuning. Conversely, the use of vendor‐provided machine data may help avoid misadministration of the beam data during data collections or data input,^7^ as well as potentially shortening the time needed for machine installation or replacement.

Varian Medical Systems provides representative beam data (RBD) for its TrueBeam^™^ linear accelerators, including most of the scanning and nonscanning data required for beam modeling for the Eclipse^™^ TPS (Varian Medical Systems). For the use of the RBD to be valid for clinical practice, any variations in the machine characteristics of recent linear accelerators must be small. However, the RBD were based on the mean data from three TrueBeam linear accelerators at one institution,^8^ and they do not contain any information about the degree of difference in the data of these three machines. Although a few studies have reported the variation of beam data of TrueBeam machines, the numbers of machines included in these studies were small.^9^, ^10^

We have established a working group to investigate variations in beam data collected in our country. In this study, we investigated the variation in TrueBeam beam data collected at multiple institutions and evaluated the difference between the RBD provided by the vendor and the beam data obtained in this study.

## 2 MATERIALS AND METHODS

We collected scanning and nonscanning data for the photon beams for 21 TrueBeam machines from 17 institutions with their agreement. The photon energies were 4, 6, and 10 MV for flattened beams and 6 and 10 MV for flattening filter‐free (FFF) beams. We only evaluated the data measured in ionization chambers with similar sensitive volumes, resulting in a limited number of datasets for small fields; Table 1 summarizes the number of data sets for each photon energy analyzed in this study. The data were collected for the beam modeling of the Eclipse TPS and were submitted by the institutions either in the scanning phantom's format or in W2CAD format generated from the scanning raw data and in the institution's administration format generated from the nonscanning raw data for the Eclipse TPS modeling. The scanning data were resampled to obtain data with a resolution of 1 mm, and the average data across the 21 machines were calculated. The vendor's RBD, generated by averaging the commissioning beam data for three TrueBeam machines, were available in W2CAD format from the vendor's website.^11^ The scanning data for the RBD and for this study were all acquired with a source‐to‐surface distance (SSD) of 100 cm. The nonscanning data for the RBD and for this study were acquired with SSDs of 95 and 90 cm, respectively. However, the only one institution's nonscanning data were acquired with an SSD of 95 cm. The types of ionization chamber used for the data collection were either CC13 (IBA Dosimetry GmbH, Schwarzenbruck, Germany) or PTW31010 (PTW, Freiburg, Germany), both with effective volumes >0.125 cm^3^. A CC13 was used to collect the source data for the RBD.

**Table 1:** The number of (a) scanning and (b) nonscanning beam data sets collected for each beam type and field size

(a) Field sizes (mm^2^)	PDD at each field sizes (mm^2^)				OCR at each field sizes (mm^2^)			
	30 × 30	100 × 100	200 × 200	300 × 300	30 × 30	100 × 100	200 × 200	300 × 300
4 MV	12	16	15	15	7	13	15	15
6 MV	12	17	16	15	7	14	16	15
10 MV	12	17	16	15	7	14	16	15
6 MV FFF	11	15	14	14	6	12	14	14
10 MV FFF	10	14	13	13	6	12	13	13

(b) Energy (MV)	OPF at each field sizes (mm^2^)					
	30 × 30	40 × 40	50 × 50	150 × 150	200 × 200	300 × 300
4 MV	14	15	8	13	15	16
6 MV	14	15	8	13	15	16
10 MV	14	15	8	13	15	16
6 MV FFF	13	14	8	12	14	15
10 MV FFF	13	13	8	12	14	13

PDD, percent depth dose; OCR, off‐center ratio; FFF, flattening filter‐free beam; OPF, output factor.

All the collected data and the vendor's RBD were imported to an Akilles RT (RADLab Inc., Osaka, Japan) software to create a database and to analyze the data. For this study, we analyzed the percent depth dose (PDD) collected at the central axis of the beam, the off‐center ratio (OCR) along the cross‐plane axis of the open field, collected at the depth of the maximum dose (*d*_MAX_) and at 10 cm depth (*d*_10_), and the output factor (OPF) of the open square field, collected at *d*_10_ and normalized according to the field size of 100 × 100 mm^2^. However, the OPF with an SSD of 95 cm was collected at *d*_5_. The field sizes of the PDD and OCR were 30 × 30, 100 × 100, 200 × 200, and 300 × 300 mm^2^ and those of the OPF were 30 × 30, 40 × 40, 50 × 50, 150 × 150, 200 × 200, and 300 × 300 mm^2^. The PDD data were normalized according to *d*_10_ to eliminate the effects of the noise around the peak. To focus on the variation in the shape of the OCR profiles, shifts in the OCR were corrected by calculating the center of the full width at half maximum. The OCR data for the flattened beams were normalized according to the value at the central axis. For FFF beams, renormalization was needed to identify the penumbra regions. The renormalization factors provided by Fogliata et al.^12^ were used to normalize the profile of the FFF beams. To compare all OPF data at d_5_ with an SSD of 95 cm, the OPF data acquired at *d*10 were corrected by a tissue‐phantom ratio (TPR)_5, 10_ of respective field sizes. The TPR data were generated by the PDD data acquired at one institution participating in this study using an OmniPro software (IBA Dosimetry). After correction, all OPF data were renormalized according to the field size of 100 × 100 mm^2^.

The dose difference (DD) and the distance‐to‐agreement (DTA) from the average beam data were used to investigate the variation in the collected scanning data. Because the DD values were calculated by subtracting the average data, each value represented the difference relative to *d*_10_ of PDD or the value at the central axis of the OCR. For the PDD data, the exponential region was evaluated using the DD. For the OCR, the field and penumbra regions were evaluated using the DD and DTA, respectively. The field region was defined as the 80% of the field size; the definition of the penumbra has been described elsewhere.^13^ Standard deviations (SD) were calculated at each data point and the maximum SD value (SD_MAX_) of the DD was calculated in the exponential region of the PDD or the field region of the OCR. The SD_MAX_ of the DTA was also calculated for the penumbra regions. The PDD data were normalized according to the value of *d*_MAX_ to evaluate the variation of the PDD_10_, which represents the quality of the photon beams. The RBD were also resampled with a resolution of 1 mm, and the difference between the RBD and the average data collected in this study were evaluated by calculating DD and DTA. The relative difference of each data with the average data collected in this study was calculated to investigate the variation in the OPF data, and the difference between the RBD and the average study data was also evaluated by calculating the relative difference.

## 3 RESULTS

**Figure 1:**
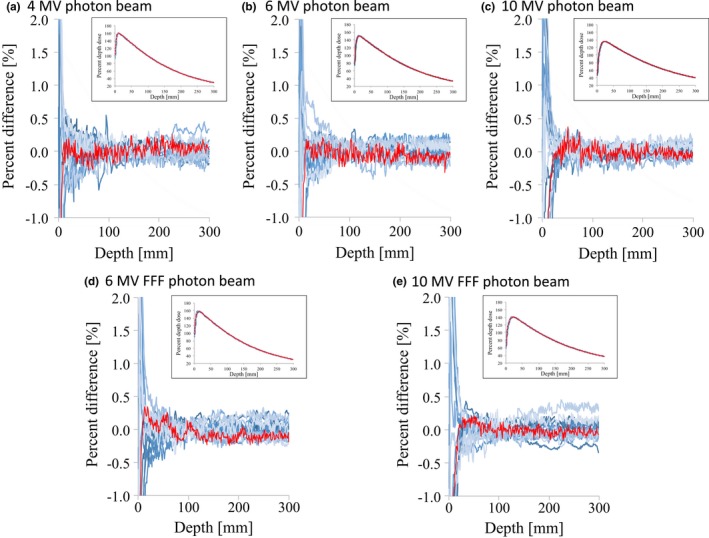
PDD curves and the dose difference between each curve and the average data for (a) 4 MV, (b) 6 MV, (c) 10 MV, (d) 6 MV FFF, and (e) 10 MV FFF photon beams with 100 × 100 mm^2^ field size. The red lines represent the vendor's RBD data. Abbreviations: PDD, percent depth dose; RBD, representative beam data.

**Figure 2:**
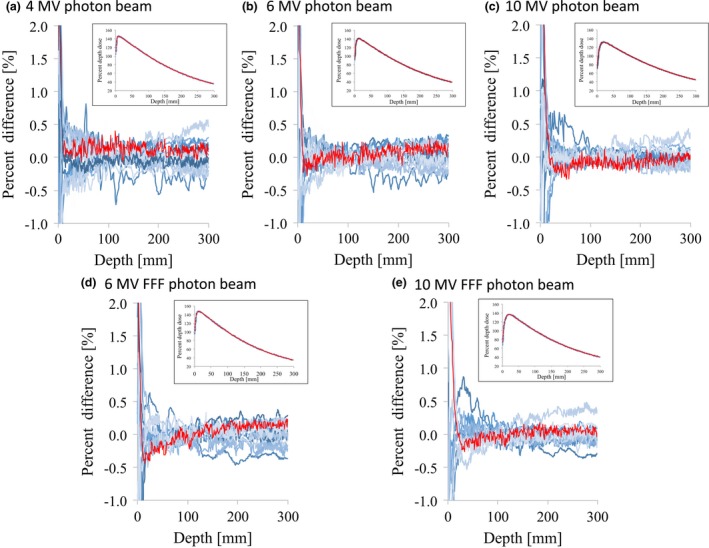
PDD curves and the dose difference between each curve and the average data for (a) 4 MV, (b) 6 MV, (c) 10 MV, (d) 6 MV FFF, and (e) 10 MV FFF photon beams with 300 × 300 mm^2^ field size. The red lines represent the vendor's RBD data. Abbreviations: PDD, percent depth dose; FFF, flattening filter‐free beam; RBD, representative beam data.

Figs. 1 and 2 illustrate the PDDs of the flattened and FFF beams, respectively, showing representative PDDs with a field size of 100 × 100 and 300 × 300 mm^2^ with a plot of the DD values. In the exponential regions, variations of the PDD were small and the DD values with field sizes of 100 × 100 and 300 × 300 mm^2^ were <1.5% and 1.0% for all energies, respectively. Similar results were obtained with field sizes of 30 × 30 and 200 × 200 mm^2^, and the maximum DD values for these were <2.0% and <1.0%, respectively. The SD_MAX_ of the DD and the maximum DD between the RBD and the average study data are summarized in Table 2. For all the energies, the SD_MAX_ of the DD were <1.0%, <0.5%, <0.5%, and <0.5% for field sizes of 30 × 30, 100 × 100, 200 × 200, and 300 × 300 mm^2^, respectively. For all energies, the values of DD between the RBD and the average study data were <1.0%, <0.5%, <0.5%, and <0.5% for field sizes of 30 × 30, 100 × 100, 200 × 200, and 300 × 300 mm^2^, respectively. We also evaluated the variation in PDD_10_ with normalizing data according to the peak value; for all energies and field sizes, the SD of the PDD_10_ were within 0.5% (data not shown).

**Table 2:** Analysis of the percent depth dose. Results are presented as the SD_MAX_ of the dose difference and the maximum dose difference between the vendor's RBD and the study data average

Field sizes (mm^2^)	SD_MAX_				Maximum DD (RBD, average)			
	30 × 30	100 × 100	200 × 200	300 × 300	30 × 30	100 × 100	200 × 200	300 × 300
4 MV	0.51	0.43	0.32	0.41	−0.46	−0.29	−0.40	0.40
6 MV	0.74	0.38	0.25	0.28	0.81	−0.32	−0.23	0.28
10 MV	0.47	0.26	0.22	0.30	0.47	0.37	0.40	−0.33
6 MV FFF	0.80	0.45	0.41	0.31	0.83	0.35	−0.31	−0.44
10 MV FFF	0.76	0.27	0.27	0.32	0.41	0.21	0.27	−0.26

Values are given in percentages.

SD_MAX_, maximum standard deviation; DD, dose difference; RBD, representative beam data; FFF, flattening filter‐free beam.

**Figure 3:**
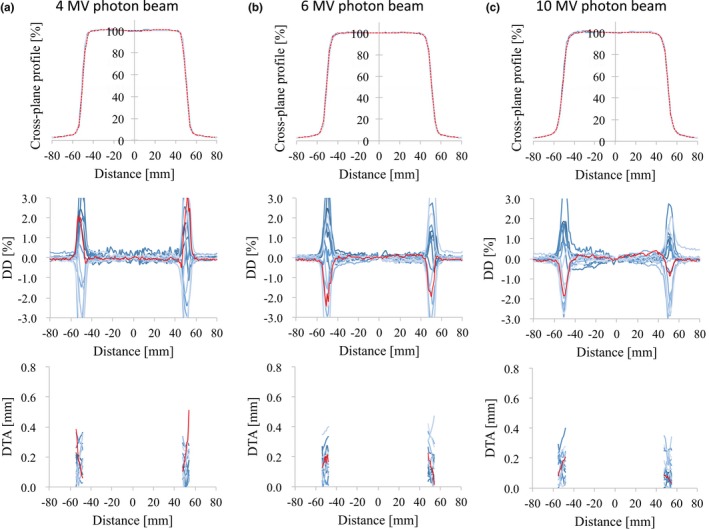
OCR profiles (upper row), the dose difference between each curve and the average data (middle row), and the distance‐to‐agreement from the average data (bottom row) for (a) 4 MV, (b) 6 MV, and (c) 10 MV photon beams with 100 × 100 mm^2^ field size. The red lines represent the vendor's RBD data. Abbreviations: OCR, off‐center ratio; RBD, representative beam data.

**Figure 4:**
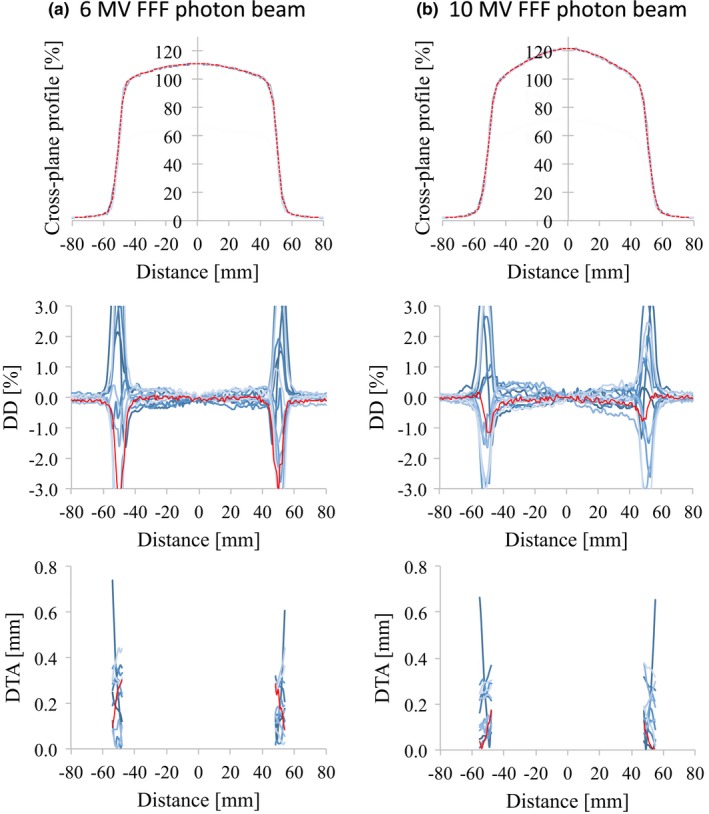
OCR profiles (upper row), the dose difference between each curve and the average data (middle row), and the distance‐to‐agreement from the average data (bottom row) for (a) 6 MV FFF and (b) 10 MV FFF photon beams with 100 × 100 mm^2^ field size. The red lines represent the vendor's RBD data. Abbreviations: OCR, off‐center ratio; FFF, flattening filter‐free beam; RBD, representative beam data.

**Figure 5:**
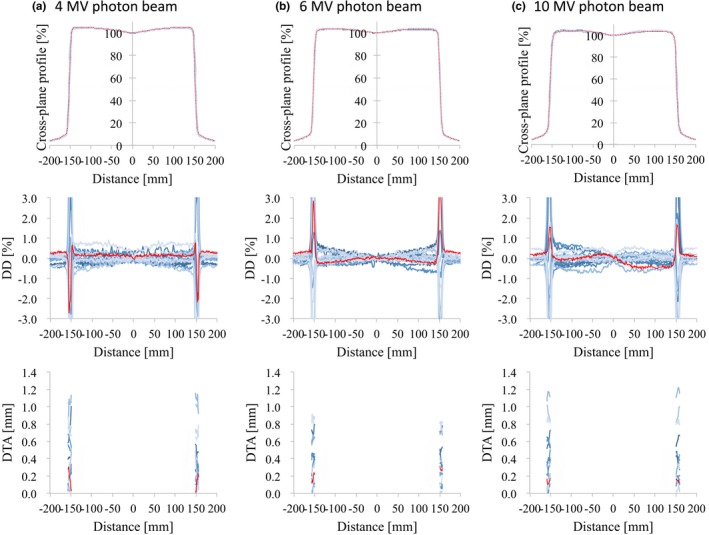
OCR profiles (upper row), the dose difference between each curve and the average data (middle row), and the distance‐to‐agreement from the average data (bottom row) for (a) 4 MV, (b) 6 MV, and (c) 10 MV photon beams with 300 × 300 mm^2^ field size. The red lines represent the vendor's RBD data. Abbreviations: OCR, off‐center ratio; RBD, representative beam data.

**Figure 6:**
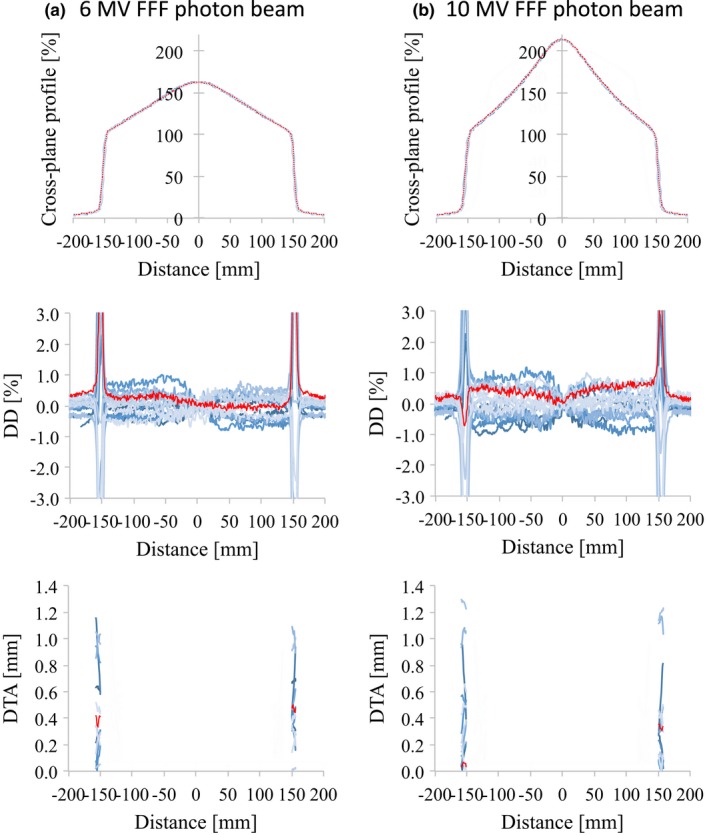
OCR profiles (upper row), the dose difference between each curve and the average data (middle row), and the distance‐to‐agreement from the average data (bottom row) for (a) 6 MV FFF and (b) 10 MV FFF photon beams with 300 × 300 mm^2^ field size. The red lines represent the vendor's RBD data. Abbreviations: OCR, off‐center ratio; FFF, flattening filter‐free beam; RBD, representative beam data.

**Figure 7:**
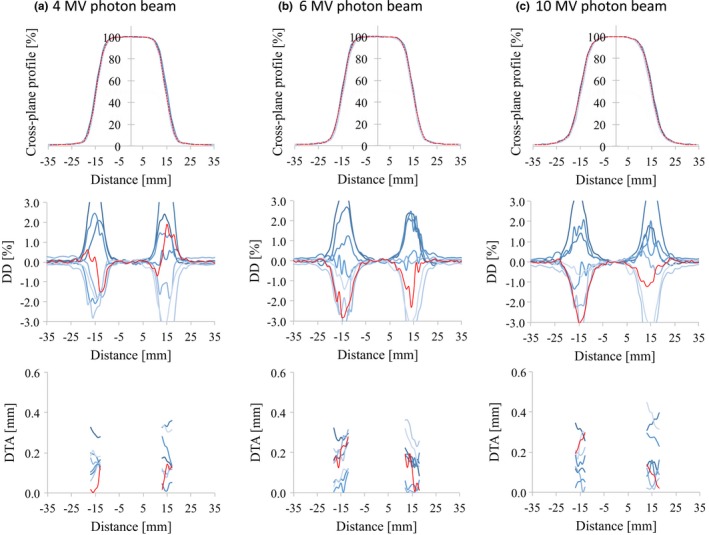
OCR profiles (upper row), the dose difference between each curve and the average data (middle row), and the distance‐to‐agreement from the average data (bottom row) for (a) 4 MV, (b) 6 MV, and (c) 10 MV photon beams at *d*_MAX_ with 30 × 30 mm^2^ field size. The red lines represent the vendor's RBD data. Abbreviations: OCR, off‐center ratio; *d*_MAX_, dose maximum; RBD, representative beam data.

**Figure 8:**
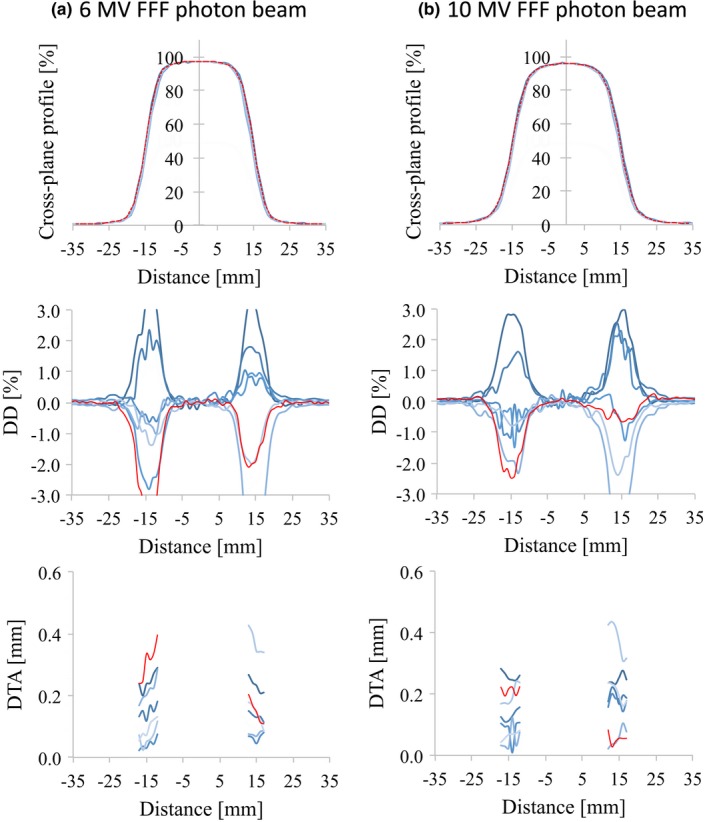
OCR profiles (upper row), the dose difference between each curve and the average data (middle row), and the distance‐to‐agreement from the average data (bottom row) for (a) 6 MV FFF and (b) 10 MV FFF photon beams at *d*_MAX_ with 30 × 30 mm^2^ field size. The red lines represent the vendor's RBD data. Abbreviations: OCR, off‐center ratio; FFF, flattening filter‐free beam; *d*_MAX_, dose maximum; RBD, representative beam data.

Figs. 3, 4, 5, 6 illustrate the OCRs of the flattened and FFF beams, respectively, showing representative d_MAX_ data for a field size of 100 × 100 and 300 × 300 mm^2^. Tables 3 and 4 summarize the values obtained for the SD_MAX_ of the DD and the maximum DD between RBD and the average study data for the OCR measured at *d*_MAX_ and *d*_10_. The profiles of the different linear accelerators showed good agreement. The DD values and the SD of the DD were <1.5% and 1.0%, respectively, for all energies and for field sizes of 100 × 100 and 300 × 300 mm^2^. For all the energies and field sizes, the SD_MAX_ of the DTA values were <0.5 mm. Similar results were found for d_10_ and the 200 × 200 mm^2^ field size. The values of DD between the RBD and the average study data were <1.0% for all energies and for field sizes of 100 × 100, 200 × 200, and 300 × 300 mm^2^. The maximum DTA values were <0.5 mm for all energies and field sizes. However, the OCR for a field size of 30 × 30 mm^2^ showed large variations for all energies (Figs. 7 and 8). Because the flattened region of a field as small as this is <80% of the full width at half maximum, some cases showed DD values >3.0%.

**Table 3:** Analysis of the off‐center ratio at *d*_MAX_. Results are presented as the SD_MAX_ of the dose difference and the maximum dose difference between the vendor's RBD and the study data average

Field sizes (mm^2^)	SD_MAX_				Maximum DD (RBD, average)			
	30 × 30	100 × 100	200 × 200	300 × 300	30 × 30	100 × 100	200 × 200	300 × 300
4 MV	2.32	0.23	0.29	0.36	−1.48	−0.14	−0.19	0.26
6 MV	2.27	0.23	0.30	0.32	−2.15	0.17	0.28	−0.26
10 MV	2.13	0.28	0.31	0.36	−2.13	0.40	0.61	−0.55
6 MV FFF	2.33	0.31	0.47	0.43	−3.13	−0.16	−0.29	0.48
10 MV FFF	2.01	0.34	0.55	0.61	−1.56	−0.25	−0.46	0.74

Values are given in percentages.

*d*_MAX_, dose maximum; SD_MAX_, maximum standard deviation; DD, dose difference; RBD, representative beam data; FFF, flattening filter‐free beam.

**Table 4:** Analysis of the off‐center ratio at *d*_10_. Results are presented as the SD_MAX_ of the dose difference and the maximum dose difference between the vendor's RBD and the study data average

Field sizes (mm^2^)	SD_MAX_				Maximum DD (RBD, average)			
	30 × 30	100 × 100	200 × 200	300 × 300	30 × 30	100 × 100	200 × 200	300 × 300
4 MV	1.48	0.22	0.29	0.32	−0.73	−0.11	−0.25	0.36
6 MV	1.44	0.20	0.28	0.27	−1.50	0.16	0.22	−0.17
10 MV	1.48	0.26	0.29	0.31	−1.21	0.23	0.39	−0.52
6 MV FFF	1.21	0.24	0.43	0.37	−1.81	−0.20	−0.21	0.42
10 MV FFF	1.44	0.34	0.50	0.50	−0.94	−0.38	−0.38	0.77

Values are given in percentages.

*d*_10_, dose at 10 cm depth; SD_MAX_, maximum standard deviation; DD, dose difference; RBD, representative beam data; FFF, flattening filter‐free beam.

**Figure 9:**
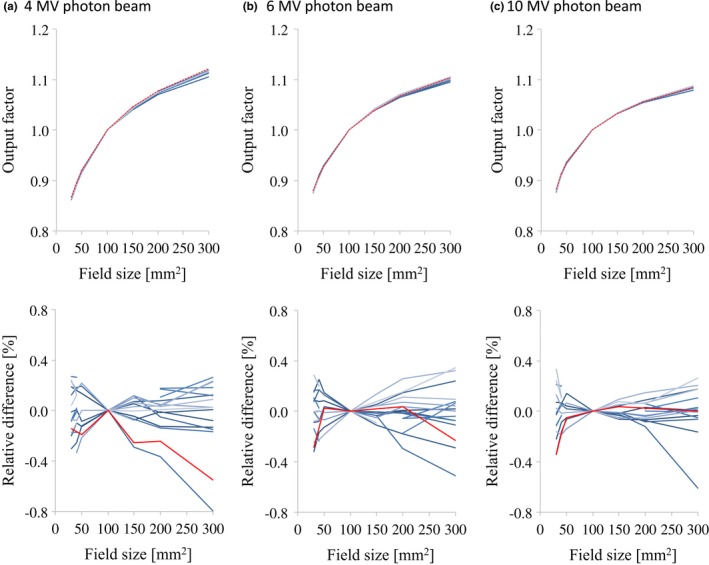
OPF curves (upper row) and the relative difference between each curve and the average data (lower row) for (a) 4 MV, (b) 6 MV, and (c) 10 MV photon beams with field sizes range from 30 × 30 mm^2^ to 300 × 300 mm^2^. The red lines represent the vendor's RBD data. Abbreviations: OPF, output factor; RBD, representative beam data

**Figure 10:**
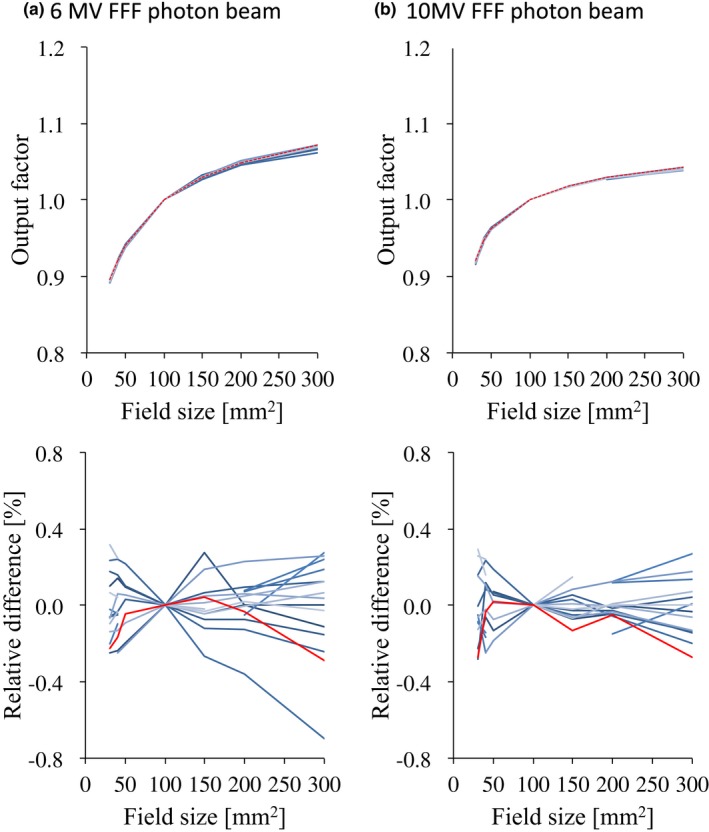
OPF curves (upper row) and the relative difference between each curve and the average data (lower row) for (a) 6 MV FFF and (b) 10 MV FFF photon beams with field sizes range from 30 × 30 mm^2^ to 300 × 300 mm^2^. The red lines represent the vendor's RBD data. Abbreviations: OPF, output factor; FFF, flattening filter‐free beam; RBD, representative beam data.

Figs. 9 and 10 illustrate the OPFs of the flattened and FFF beams, respectively, with a plot of the relative difference. For all energies and field sizes, the OPF data of the different linear accelerators showed good agreement and the relative differences of each data with the average study data were <1.0%. The maximum relative differences between the RBD and the average study data were <1.0% for all energies and field sizes.

## 4 DISCUSSION

In this study, we collected scanning and nonscanning beam data of TrueBeam linear accelerators from multiple institutions and analyzed the differences between them. The PDD data showed only very small variations. In the exponential regions, the SD_MAX_ of the DD and the SD of the PDD_10_ were <1.0% and 0.5%, respectively. Cho et al.^14^ investigated the linear accelerators of more than 50 institutions and reported that the SD for the measured PDD values was <1.0%. Glide‐Hurst et al.^9^ compared the PDDs of five TrueBeam machines and reported that the variation in PDD_10_ was within 0.3% for photon beams of 6, 10, and 15 MV. Beyer et al.^10^ also reported <1.0% variability in PDD_10_ for three TrueBeam machines. Our results confirmed the small variation in the PDD of TrueBeam machines with data from multiple linear accelerators.

The OCR data for field sizes of 100 × 100, 200 × 200, and 300 × 300 mm^2^ also showed only small variations. For field sizes greater than 300 × 300 mm^2^, often, data are collected with phantom offsets and include an uncertainty in the mirroring process of half profiles. However, profiles collected with and without phantom offsets should match within 0.5%,^13^ and this measurement for large field sizes was used for the RBD large profiles. Beyer et al.^10^ reported that variations in photon beam profiles between three TrueBeam machines were <1.0% in the low gradient area, and that the gamma pass rate was 100% for the criterion of 2.0%/1.0 mm. Chang et al.^8^ showed that the mean SD of the profiles of three TrueBeam machines was 0.4% for 10‐MV FFF open fields.

The OPF data for field sizes ranging from 30 × 30 to 300 × 300 mm^2^ also showed very small variations, and the relative differences of each data with the average study data were less than 1.0% for all energies and field sizes. Cho et al.^14^ reported that the SD for the measured OPF values was always <1.0% among the linear accelerators of more than 50 institutions. Glide‐Hurst et al.^9^ showed that the largest coefficient of variation in the OPFs among five TrueBeam machines was 0.5%.

For field sizes of 100 × 100, 200 × 200, and 300 × 300 mm^2^, the values of PDD and OCR for the average study data both showed good agreement with those for the vendor's RBD. The maximum values of DD between the RBD and the average study data were <0.5% and 1.0% in the exponential regions of PDD and in the field regions of OCR, respectively. The maximum DTA values of the OCR were <0.5 mm. Moreover, the OPF values for the average study data showed good agreement with those for the vendor's RBD. The relative differences between the RBD and the average study data were <1.0% for all energies and field sizes. The values of the source OPF data for the average study data were based on the mean data from the corrected OPF by an approximate expression of the values at *d*_5_ of the one institution's TPR normalized according to *d*_10_. However, this uncertainty is considered to be tiny because of the results that variations of the collected PDD were very small. These findings supported the RBD as being representative of TrueBeam machines.

However, for the 30 × 30 mm^2^ field size, there were large SD values of DDs (≥2.0%) in the field regions. When the field size is this small, the field region contains the horn regions of the OCR. Although all the institutions used ionization chambers with similar cavity volumes, various factors can affect the measured profile, including the direction of the chamber settings, the data collection mode (step‐by‐step or continuous), the duration of the measurement at each point, and the dose rate. Although some positions showed a DD of 1.0% or more in the field region, the DTA values at these positions were <0.5 mm. The SD_MAX_ of the DTA in the penumbra region was also <0.5 mm for all the energies and field sizes. When using detectors for small field dosimetry, such as a diode or diamond detector, the variations will become much larger because the penumbra measured with these detectors will be much steeper than that measured with ionization chambers.^4^, ^15^ In addition, the scanning water phantom systems used for the measurements were not the same for all institutions. However, as reported by Akino et al.^16^, the effect of the scanning phantom would be expected to be negligible. As CC13 was used to collect the source data for the RBD, including small field and FFF beam data^17^, we have to measure data by using it comparing with the RBD. Data measurement with other suitable devices, such as a smaller cavity chamber, diode, and diamond detector, might be needed for accurate commissioning of small field and FFF beam data; however, the RBD do not include these data. Furthermore, larger variations in beam profiles for small field dosimetry could be caused by machine characteristics that are based on machine design, beam generation, jaw positional accuracy, and beam focal spot size differences.^18^ Although the accuracy of the multileaf collimator position is <1 mm at isocenter, jaw positional accuracies are within 2 and 1 mm for the upper and lower jaws, respectively, according to specifications provided by the vendor. The accuracy of beam modeling and the commissioning of TPSs for small field beam data are very important for the implementation of advanced radiotherapy techniques. These are the limitations for the use of the RBD in clinical practice.

In its report on accelerator beam data commissioning, the Task Group 106^4^ of the Therapy Physics Committee of the American Association of Physicists in Medicine recommended that the measurement error should be reduced below ±1.0% by using suitable devices and methods. Except for small field dosimetry, our results demonstrated this criterion had been met, indicating that the intermachine variability of the TrueBeam linear accelerators was very small and supporting the validity of using the vendor's RBD for clinical applications. The use of the RBD might greatly facilitate the quick installation of the linear accelerator.

## 5 CONCLUSIONS

We evaluated the variation in the PDD, OCR, and OPF values of 21 TrueBeam linear accelerators. The variations from the average study data were very small, representing the low variability in the manufacture of recent accelerators. The average of the data obtained in this study showed very similar PDD, OCR, and OPF curves to those of the RBD provided by the linear accelerator vendor.

## CONFLICT OF INTEREST

One of the authors (YA) is a developer of the software Akilles RT, which was used in this study.
